# Ethnic Differences in Insulin Resistance, Adiponectin Levels and Abdominal Obesity: Haitian Americans and African Americans, with and without Type 2 Diabetes Mellitus

**DOI:** 10.9734/bjmmr/2014/10333

**Published:** 2014-06-16

**Authors:** Amanpreet K. Cheema, Gustavo G. Zarini, Joel Exebio, Sahar Ajabshir, Lemia Shaban, Janet Antwi, Joan A. Vaccaro, Fatma G. Huffman

**Affiliations:** 1 Department of Dietetics and Nutrition, Robert Stempel College of Public Health, Florida, International University, Miami, USA.; 2 Department of Food Science and Nutrition, College for Life Sciences, Kuwait University, Kuwait City, Kuwait.

**Keywords:** Type 2 Diabetes Mellitus, Hs-CRP, adiponectin, insulin resistance, HOMA2-IR, A1C, Haitian Americans, African Americans

## Abstract

**Background:**

Metabolic outcomes of obesity and its associated disorders may not be equivalent across ethnicity and diabetes status.

**Aim:**

In this paper, we examined the association of abdominal obesity, by ethnicity and diabetes status, for indicators of glucose metabolism in Blacks.

**Methods:**

A cross sectional study was conducted in Haitian Americans (n= 186) and African Americans (n= 148) with and without type 2 diabetes mellitus (T2DM). Student’s t-test and Chi-squared test were used to assess differences in mean and proportion values between ethnicities with and without type 2 diabetes mellitus. Relationship between insulin resistance, ethnicity, diabetes status, abdominal obesity, and adiponectin levels were analyzed by analysis of covariance while controlling for confounding variables.

**Results:**

Haitian American participants were older (*P* = .032), had higher fasting plasma glucose (*P* = .036), and A1C (*P* = .016), but had lower levels of Hs-CRP (*P* < .001), insulin and HOMA2-IR and lower abdominal obesity (*P* = .030), than African Americans. Haitian Americans had significantly lower HOMA2-IR (*P* = .008) than African Americans when comparing both ethnicities with T2DM, high abdominal obesity, and adiponectin levels lower than the median (<14.75 ng/mL).

**Conclusion:**

The clinical significance of observed differences in insulin resistance, abdominal obesity, and adiponectin levels between Haitian Americans and African Americans could assist in forming public health policies that are ethnic specific.

## 1. INTRODUCTION

Obesity increases the chances of developing serious health conditions, such as insulin resistance and type 2 diabetes mellitus (T2DM) [1, 2]. According to a recent study, by the year 2025, the number of individuals with diabetes will increase by 64% [3]. Obesity corresponds to an increase in adipose tissue mass, which is associated with alterations in the metabolic and endocrine functions of adipocytes [4]. Adiponectin is one of the many adipokines released by adipocytes that are involved in regulation of insulin sensitivity [5] and a variety of metabolic processes including energy homeostasis, lipid and glucose metabolism [6]. It has been suggested that high adiponectin levels protect against the development of insulin resistance in healthy individuals [7–8], and high adiponectin levels are inversely associated with abdominal obesity [9–10]. Increasing abdominal adiposity decreases mRNA expression for adiponectin, and results in low levels of adiponectin as indicated in several observational studies, in Japanese [11], Pima Indians [12] and Asian Indians [13–14].

Low levels of adiponectin have been associated with insulin resistance, risk for cardiovascular disease, and T2DM [15–16]. Insulin resistance develops when insulin becomes less efficient in lowering blood sugar [17]. Insulin resistance is a risk factor for T2DM and has been associated with high body mass index (BMI) [18]. Moreover, abdominal obesity as measured by waist circumference (WC) is considered a stronger independent risk marker for insulin resistance than overall adiposity [19–21]. Insulin resistance as well as adiponectin level has been shown to differ by ethnicity [22–24]. Individuals of African origin which include African Americans and Caribbean populations, such as Haitian Americans and Jamaican Americans, are considered high risk groups for T2DM [25]. Prevalence of abdominal obesity, hypertension and T2DM is higher in populations with African than European descent [26–27].

Desilets et al. [28] reported low insulin resistance in Haitian Americans as compared to White Americans with comparable abdominal obesity. Their results were contrary to studies which suggested that African Americans are more insulin resistant than White Americans [29–31]. According to Vimalananda et al. [32], despite the worse glycemic control, poor control of LDL cholesterol and blood pressure found in Haitian Americans, rates of microvascular as well as macrovascular complications were lower in Haitian Americans than African Americans or non-Hispanic Whites with T2DM. This may suggest that Haitian Americans do not have the same clinical characteristics as African Americans; therefore, results found in African Americans cannot be extrapolated to Haitian Americans [33–34]. Furthermore, failure to acknowledge differences in metabolic profiles of the two groups could have devastating effects on the control of T2DM and its related complications. Few studies have investigated the associations among WC, adiponectin, diabetes status and insulin resistance, and they have been primarily in White populations [35,20]. Based on the gap in the literature concerning insulin resistance, adiponectin, obesity, ethnicity and diabetes status, within the Black population, the present study was conducted. The objective of the study was to assess ethnic differences in insulin resistance, adiponectin levels, and abdominal obesity in individuals with and without type 2 diabetes mellitus, within the Black community in South Florida.

## 2. METHODS AND PARTICIPANTS

### 2.1 Participants and Study Design

This was a cross-sectional study, conducted with Haitian Americans and African Americans with and without T2DM. Recruitment was conducted by alternating between selecting potential participants with and then without T2DM. The participants were initially recruited from randomly generated mailing lists. The lists of addresses were purchased from Knowledge Base Marketing, Inc., Richardson, TX 75081. This company provided two mailing lists generated from multiple databases of African Americans, identified as having or not having T2DM, from Miami-Dade and Broward Counties, Florida. During a one year period, approximately 7,550 letters, mentioning outline of the study were mailed to African Americans with and without T2DM. Approximately, 6.3% (n=477) of the letters were returned due to unknown addresses. From the remaining delivered letters, 4.0% (n=256) responded. We were not able to recruit Haitian Americans in the same manner because Knowledge Base Marketing, Inc. did not have a database for the Haitian Americans community. Therefore, recruitment of Haitian American participants (n=259) were from community-based sources: (a) Local diabetes educators & community health practitioners in Miami-Dade and Broward Counties: official letters mentioning outline of the study were mailed to all diabetic educators and health professionals in Miami-Dade and Broward counties, requesting their cooperation in recruiting individuals; (b) Florida International University (FIU) faculty, staff and students: invitational flyers were distributed to all university faculty, staff and students explaining the research protocol and requesting their assistance in the study; (c) Print advertisements were placed in local Haitian American newspapers and principal gathering places, churches, supermarkets, and restaurants; (d) Radio advertisement on local Creole station was also aired.

This study was approved by the Institutional Review Board at FIU. Interested participants were initially interviewed telephonically. The purpose of the study was explained and basic information like age, gender was noted. To ascertain T2DM status, each participant who self-reported having diabetes was asked for the age of diagnosis, and initial treatment modalities. If a subject was determined to be eligible, then his or her participation was requested at the Human Nutrition Laboratory at FIU. Participants were instructed to refrain from smoking, consuming any food or beverages except water, and engaging in any unusual exercise for at least eight hours prior to their blood collection. The purpose and protocol of the study was explained to the participants, and their written consent either in English or Creole was obtained prior to the commencement of the study. Laboratory results showed that twelve participants (Haitian Americans = 8; African Americans = 4) who reported not having diabetes were reclassified as having T2DM according to American Diabetes Association standards (ADA). Participants were given their laboratory results and referred to their physicians. A total of 334 participants (Haitian Americans=186 and African Americans=148) were included in the data analysis.

### 2.2. Data Collection

#### 2.2.1 Procedure

Participants were asked to fill out standard questionnaires on site. Trained interviewers who were bilingual in English and Creole were present to administer the questionnaires. Through administration of questionnaires, information was obtained on participants’ sociodemographic data, smoking history and status, and medications usage.

#### 2.2.2 Anthropometric measurements

Height and weight were measured using a SECA balance scale (Seca Corp, MD, US). BMI was calculated as weight in kg/height in m^2^. Obesity was defined as having BMI ≥ 30 kg/m^2^ [36]. Waist circumference (WC) to the nearest 0.1 cm was measured horizontally with a non-stretchable measuring tape placed midway between the 12th rib and iliac crest at minimal respiration to determine abdominal obesity (male = 102 cm/ female = 88 cm) [36].

### 2.3 Blood Collection

Venous blood was collected from each participant after an overnight fast (at least 8 hours) by a certified phlebotomist using standard laboratory techniques. Two different blood samples were collected: 1) serum glucose determination (Vaccutainer Serum Separator tube), and 2) for glycosylated hemoglobin (A1C, via EDTA collection tube). Glucose levels were measured by conventional hexokinase enzymatic methods with inter coefficient of variation (CV) = 2.2% and intra CV=10.1%. Serum total adiponectin was measured using an enzyme-linked immunosorbent assay (ELISA) with inter and intra CV to be 8.4% and 7.4% respectively (Linco Reearch Inc, MO, US). Percentages for A1c were measured from whole blood samples using Roche Tina Quant method by a certified clinical laboratory with both intra and inter CV=3.57% (Laboratory Corporation of America, LabCorp, FL, US). Serum insulin levels were determined using the Human Insulin ELISA kit from Millipore (St Charles, MZ, U.S.). High-sensitivity C-reactive protein (Hs-CRP) was analyzed in serum using Immulite method with inter CV=5.4% and intra CV=8.2%. A 1:100 manual dilution of the antibody provided a measurable range of 0.1–500 mg/L [37].

### 2.4 Determination of Insulin Resistance (HOMA2-IR)

Insulin resistance index was determined using the Oxford University HOMA2 calculator [38] from paired fasting plasma glucose and radioimmunoassay insulin, across a range of 1–25 mmol/l for glucose, and 1–2,200 pmol/l for insulin [38–39].

### 2.5 Statistical Analysis

Prior to analysis, all variables were tested using the Kolmogorov-Smirnov test for normality. Variables Hs-CRP and HOMA2-IR were log-transformed. Differences in mean values of biochemical indicators by ethnicities and diabetes status were determined using the Student’s t-test and analysis of variance (ANOVA). A post-hoc analysis was performed to determine differences among diabetes by ethnicity. Chi-squared test was used to determine differences in categorical variables according to ethnicity and diabetes status. Median adiponectin was used to create a categorical variable since there was no established clinical cut off point for adiponectin. Analysis of covariance (ANCOVA) was used to determine the relationship between the dependent variable HOMA2- IR and the independent variables ethnicity (Haitian Americans, African Americans), diabetes status (yes/no), abdominal obesity (male = 102 cm/ female = 88 cm) [36], adiponectin (14.75 ng/mL as median) and their interactions. There is no established clinical cutoff range for adiponectin levels; therefore, median (14.75 ng/mL) was used as cutoff to compare groups. The analysis was adjusted for age, gender (male/female), smoke (yes/no), nonsteroidal anti-inflammatory drugs (NSAIDs) (yes/no) and log Hs-CRP. All analyses used SPSS version 18 (SPSS Inc., Chicago, IL, US) and *P* values < .05 were considered statistically significant.

## 3. RESULTS

For this study, comprehensive data was available for Haitian Americans (n=186) and African Americans (n=148), and 40% of both ethnicities had T2DM. The general characteristics of study population by ethnicity are described in Table 1.

Haitian Americans, as compared to African Americans were older (mean age 55.7±10.8), had higher fasting plasma glucose and A1C. African Americans had larger BMI (*P* < .00l), WC (*P* < .001), and higher percentage of abdominal obesity (*P* = .030). African Americans also had higher insulin levels (*P* = .017), HOMA2-IR levels (*P* = .038), Hs-CRP and log-Hs- CRP (*P* < .001 and *P* = .001) than Haitian Americans. African Americans had the highest percentage of smokers (38.5%) as compared to Haitian Americans (4.8%). There were no differences in percentage of individuals taking oral anti-hyperglycemic meds or with T2DM between the two ethnicities.

The general characteristics of study population by diabetes status are described in Table 2. Participants with T2DM across two ethnicities were older (*P* < .001), had a higher percentage using oral anti-hyperglycemic medications (*P* < .001), had larger BMI (*P* = .007), larger WC (*P* < .001), and higher percentage of abdominal obesity (*P* = .002). In individuals with T2DM, higher levels of adiponectin (*P* = .038), FPG (*P* < .001), A1C levels (*P* < .001), Hs-CRP and log-Hs-CRP (*P* = .015 and *P* = .019) were also observed.

The general characteristics of participants by ethnicity and diabetes status are compared and described in Table 3. Haitian Americans with T2DM were older (*P* = .009) than Haitian Americans without T2DM and African Americans without T2DM (*P* <.001). African Americans with T2DM were older (*P* = .009) than African Americans without T2DM. Haitian Americans with T2DM were older (*P* = .009) than Haitian Americans without T2DM.

African Americans with and without T2DM had higher percentage of smokers than Haitian Americans with and without T2DM, respectively (*P* <.001). African Americans with T2DM had higher BMI and WC than African Americans without T2DM (*P* <.001) and Haitian Americans with (*P* <.001) and without (*P* <.001) T2DM. African Americans with T2DM had a higher percentage of obesity than African Americans without T2DM and Haitian Americans with and without T2DM (*P* <.001). African Americans with T2DM had higher abdominal obesity (*P* <.05) than Haitian Americans without T2DM. African Americans with T2DM had higher Hs-CRP and log Hs-CRP (*P*<.001) than Haitian Americans without T2DM. African Americans with T2DM had higher adiponectin levels than African Americans without T2DM (*P* =.003) and Haitian Americans with and without T2DM (*P* <.001). African Americans without T2DM had higher adiponectin levels than Haitian Americans with (*P* = .032) and without (*P* = .027) T2DM. Haitian Americans with and without T2DM had higher percentage of adiponectin (<14.75 ng/mL) than African Americans with T2DM (*P* <.05). Haitian Americans with T2DM had higher FPG than African Americans with (*P* = .020) and without (*P* < .001) T2DM and Haitian Americans without T2DM (*P* < .001). African Americans with T2DM had higher FPG than African Americans without T2DM (*P* < .001) and Haitian Americans without T2DM (*P* < .001). Haitian Americans with T2DM had higher A1C levels than African Americans with (*P* <.001) and without T2DM (*P* <.001) and Haitian Americans without T2DM (*P* <.001). African Americans with T2DM had higher A1C levels than African Americans without T2DM (*P* <.001) and Haitian Americans without T2DM (*P* <.001).

Haitian Americans without T2DM with normal WC and adiponectin levels (< 14.75 ng/mL) had significantly lower HOMA2-IR (*P* = .003) than African Americans. Haitian Americans with T2DM with high WC and adiponectin levels, <14.75 ng/mL also had significantly lower HOMA2-IR (*P* = .008) than African Americans Table 4 (above). Haitian Americans without T2DM and with high WC in the adiponectin <14.75 ng/mL group had higher HOMA2-IR (*P* < .001) as compared to those with normal WC. Haitian Americans with T2DM and high WC in the adiponectin >14.75 ng/mL group had higher HOMA2-IR (*P* = .035) as compared to those with normal WC. African Americans with T2DM and high WC in the adiponectin <14.75 ng/mL group had higher HOMA2-IR (*P* = .003) as compared to those with normal WC Table 5 (above).

Haitian Americans had lower insulin resistance than African Americans with comparable characteristics. In sample population with normal WC (normal WC: male = 102 cm/ female = 88 cm), adiponectin levels < 14.75 ng/mL and without T2DM, HOMA2-IR (*P* = .003) was significantly lower in Haitian Americans than African Americans. The lower HOMA2-IR (*P* = .008) in Haitian Americans was also significant when individuals of both ethnicities with T2DM, high WC and adiponectin levels <14.75 ng/mL were compared Table 4. Haitian Americans without T2DM and high WC in the adiponectin <14.75 ng/mL group had higher HOMA2-IR (*P* < .001) as compared to those with normal WC. Haitian Americans with T2DM and high WC in the adiponectin >14.75 ng/mL group had higher HOMA2-IR (*P* = .035) as compared to those with normal WC. African Americans with T2DM and high WC in the adiponectin <14.75 ng/mL group had higher HOMA2-IR (*P* = .003) as compared to those with normal WC Table 5.

## 4. DISCUSSION

Insulin resistance, adiponectin levels and abdominal obesity were compared across diabetes status of two Black ethnicities. Significant ethnic differences were found. Haitian Americans without T2DM with normal WC and low adiponectin levels (< 14.75 ng/mL) had significantly lower HOMA2-IR as compared to African Americans. Plasma adiponectin levels are shown to be lower with the development of obesity, diabetes, and metabolic disorders [40–42]. Circulating adiponectin levels decrease with increasing insulin resistance [42]. Since adiponectin levels differed by diabetes status, we examined the ethnic differences within diabetes status.

Adiponectin levels vary among ethnicities [43]. African Americans have lower plasma adiponectin levels, than their White counterparts [43–45]. Insulin resistance was directly proportional to adiponectin levels in Haitian Americans participants but this association was not present for African Americans. Haitian Americans were found to have lower insulin resistance than African Americans with and without T2DM in the presence of high and normal waist circumference (WC), when adiponectin levels were < 14.75 ng/mL. The lower prevalence of abdominal obesity in Haitian Americans than African Americans may explain the presence of low insulin resistance in Haitian Americans as compared to African Americans. This lower insulin resistance in Haitian Americans could be due to the presence of different dietary, genetic and environmental factors (lifestyle) distinguishing Haitian Americans from African Americans within Black population [43–46]. A previous study by Huffman et al. [47] reported Haitian Americans to have a better diet quality than African Americans.

The results also suggest a strong relationship between WC and insulin resistance in Haitian Americans regardless of diabetes status, as confirmed by Wahrenberg et al. and Lee et al. [20–21]. Thus, WC is considered to be a strong independent risk factor for insulin resistance. The positive relationship between WC and insulin resistance was seen in African Americans with T2DM but for individuals without T2DM, results were not significant. One possible explanation could be the presence of high insulin resistance in general, which makes it difficult to detect the difference in African Americans without T2DM. Insulin resistance increases with age but this was not seen in Haitian Americans, who were significantly older than African Americans but demonstrated lower insulin resistance, suggesting that there are other factors more significant than age in this population that play a role in developing insulin resistance. Eliasson and colleagues [48–49] found smoking to be an important contributor to insulin resistance in two different studies. This positive association between smoking and insulin resistance has also been proposed by studies in Finnish men in military service [50], and insulin resistance was shown to be aggravated in smokers with T2DM [51–52]. African Americans in our study had a higher percentage of smokers, higher BMI, larger WC, higher insulin and HOMA2-IR levels than Haitian Americans. It is suggested that higher insulin resistance could be attributed to smoking and obesity among African Americans.

There are several limitations to this study. One limitation was the cross sectional design of the study, that precluded the establishment of cause and effect. Second, the study population may not be the true representative of African Americans and Haitian Americans as general population may be different from the participants of this study. Our recruitment method was different in our African American population, using a purchased list, compared to the method used with our Haitian American population, in which community resources were employed to recruit participants. Thus, recruitment method could have influenced the type of person who responded. Another possible limitation was that total adiponectin, as oppose to isoforms, was measured. The metabolism of different isoforms may differ across ethnicity and diabetes status. However, relative differences are tested across groups rather than absolute differences in terms of adiponectin values which would have caused no bias. Insulin resistance was not measured directly, but instead we used HOMA-IR calculation to evaluate the levels of insulin resistance [38–39]. The HOMA2-IR Oxford method uses radioimmunoassay insulin for calculations whereas the insulin calculated is by using ELISA which underestimates the values than radioimmunoassay. As the calculated insulin resistance is being compared across the study using same methodology, this limitation does not have a major impact on the analysis. Moreover, there is no other calculator for insulin resistance that uses ELISA measured values of insulin. The validity of HOMA [44,53–55] and HOMA2 has been established as accepted surrogate indicator of insulin resistance [31,21,53–55].

Despite some limitations, this study demonstrated association of diabetes, abdominal obesity and adiponectin levels with insulin resistance in African Americans and Haitian Americans. No published study, to the best of our knowledge, has compared insulin resistance and adiponectin levels within Black ethnicities with and without T2DM. Significant differences in anthropometric parameters, plasma adiponectin levels and insulin resistance were found in adult African Americans and Haitian Americans, with and without T2DM adjusted for age, sex, smoking, NSAIDs and Hs-CRP, living in and around Miami, FL. These results suggest that the two ethnic groups may have different metabolic risk factors and that the guidelines for adiponectin may need to be ethnically specific.

## 5. FUTURE IMPLICATIONS

Several research studies have contributed in understanding the presence of variations in metabolic markers and genetic predispositions to certain metabolic disorders among different ethnicities. Continuing research of this nature shows a great potential for making public health professionals equipped to individualize and optimize prevention and treatment policies for different target ethnicities in the globalized world.

## 6. CONCLUSION

The study demonstrated ethnic differences in adiponectin levels in subpopulation groups of African ancestry. There are few reports of multiethnic groups and adiponectin levels, but a lot of progress has been made recently in identifying individual genetic variants associated with the adiponectin trait. Future studies should continue to assess the relationships among adiponectin, obesity and insulin resistance within individuals of Black ethnicities along with their association with the established genetic variants for adiponectin trait. Establishment of the relationship among adiponectin, abdominal obesity, and insulin resistance specific to ethnicity and diabetes status is needed to design interventions for the prevention of diabetes and diabetes complications.

## Figures and Tables

**Table 1.** Descriptive statistics by ethnicity

Variables	Haitian Americans	African Americans	*P*-value

	n=186	n=148	

Age (years)	55.7±10.8	53.2±9.5	.032
Gender(M)	89 (47.8)	81 (54.7)	.211
Diabetes status (yes)	75 (40.3)	61 (41.2)	.869
Oral anti-hyperglycemic meds (yes)	64 (34.4)	43 (29.1)	.298
Smoking (yes)	9 (4.8)	57 (38.5)	< .001
BMI (kg/m^2^)	28.9±4.8	31.9±6.6	< .001
Obesity (bMi > 30 kg/m^2^) (yes)	66 (35.5)	80 (54.1)	.001
WC (cm)	96.8±12.2	104.9±16.1	< .001
Abdominal Obesity (yes)	53.8	65.5	.030
Hs-CRP (ng/mL)	2.2±2.0	3.0±2.4	< .001
Log-Hs-CRP	0.36±.9	0.71 ±.9	.001
Adiponectin (ng/mL)	16.8±9.1	24.9±15.7	< .001
Adiponectin (<14.75 ng/mL)	92 (49.5)	42 (28.4)	< .001
FPG (mmol/L)	119.2±47.5	109.1±38.5	.036
A1C (%)	6.7±1.8	6.3±1.2	.016
Insulin (μIU/mL)	10.1 ±6.9	12.3±9.7	.017
HOMA2-IR	1.4±0.9	1.6±1.2	.038
Log-HOMA2-IR	0.15±.5	0.28±6	.057

Note: Data were expressed as mean ± SD for continuous variables or N (%) for categorical variables. Abbreviations: BMI= body mass index; WC= waist circumference; HOMA2-IR= Homeostasis Model Assessment for Insulin Resistance; Hs-CRP= high sensitivity C- reactive protein; FPG= fasting plasma glucose; A1C= hemoglobin A1C; abdominal obesity (male = 102 cm/ female = 88 cm); adiponectin (median- < 14.75 low/ ≥ 14.75 high)

**Table 2.** General characteristics of participants by diabetes status

Variables	Subjects		*P*-value

	Without T2DM n=198	With T2DM n=136	

Age (years)	52.6±9.7	57.5±10.3	< .001
Gender(m)	52.5	48.5	.473
Ethnicity			.869
Haitian American	111 (56.1)	75 (55.1)	
African American	87 (43.9)	61 (44.9)	
Oral anti-hyperglycemic meds (yes)	0 (0.0)	107 (78.7)	< .001
Smoking (yes) BMI (kg/m^2^)	41 (20.7)	25 (18.4)	.600
	29.5±5.3	31.2±6.4	.007
Obesity (BMI > 30 kg/m^2^) (yes)	75 (37.9)	71 (52.2)	.010
WC (cm)	98.1±13.2	103.8±15.9	< .001
Abdominal Obesity (yes)	103 (52.0)	94 (69.1)	.002
Hs-CRP (ng/mL)	2.3±2.1	2.9±2.3	.015
Log-Hs-CRP	0.41±.98	0.66±.9	.019
Adiponectin (ng/mL)	19.1 ±10.9	22.2±15.6	.038
Adiponectin (<14.75 ng/mL)	84 (42.4)	50 (36.8)	.300
FPG (mmol/l)	97.0±15.8	140.4±57.3	< .001
A1C (%)	5.9±.5	7.5±2.1	< .001
Insulin (μIU/mL)	11.0±7.9	11.3±8.9	.729
HOMA2-IR	1.4±1.0	1.6±1.2	.187
Log-HOMA2-IR	0.17±.5	0.26±.6	.187

Note: data were expressed as mean ± SD for continuous variables or N (%) for categorical variables. Abbreviations: BMI= body mass index; WC= waist circumference; HOMA2-IR=Homeostasis Model Assessment for Insulin Resistance; Hs-CRP= high sensitivity C- reactive protein; FPG= fasting plasma glucose; A1C= hemoglobin A1C; abdominal obesity male = 102 cm/ female = 88 cm); adiponectin (median- < 14.75 low/ ≥ 14.75 high)

**Table 3.** General characteristics of participants by ethnicity and diabetes status

Variables	Haitian Americans		African Americans	

	without T2DM n=111	with T2DM n=75	without T2DM n=87	with T2DM n=61

Age (years)	53.8±10.8^a^	58.5±10.0^b^	51.0±7.9^c^	56.4±10.7^ab^
Gender (M)	56(50.5)	33(44.0)	48(55.2)	33(54.1)
Oral anti-hyperglycemic meds (yes)	0(0)^a^	64(85.3)^b^	0(0)^a^	43(70.5)^b^
Smoking (yes)	6(5.4)^a^	3(4.0)^a^	35(40.2)^b^	22(36.1)^b^
BMI (kg/m^2^)	28.8±4.9^a^	28.9±4.6^a^	30.4±5.7^a^	34.1±7.2^b^
Obesity (BMI > 30 kg/m^2^) (yes)	38(34.2)^a^	28(37.3)^a^	37(42.5)^a^	43(70.5)^b^
WC (cm)	96.1±12.5^a^	97.8±11.7^a^	100.5±13.7^a^	111.2±17.4^b^
Abdominal Obesity (yes)	53(47.7)^a^	47(62.7)^a,b^	50(57.5)^a,b^	47(77.0)^b^
Hs-CRP (ng/mL)	2.0±1.9^a^	2.4±2.2^a^	2.7±2.4^a^	3.5±2.4^b^
Log-Hs-CRP	0.28±1.0^a^	0.48±1.0^a^	0.58±1.0^a^	0.90±1.0^b^
Adiponectin (ng/mL)	16.9±9.1^a^	16.5±9.2^a^	22.0±12.3^b^	29.1 ±18.9^c^
Adiponectin (<14.75 ng/mL)	55(49.5)^a^	37(49.3)^a^	29(33.3)^a,b^	13(21.3)^b^
FPG (mmol/l)	99.0±17.1^a^	149.1±60.8^b^	94.6±13.7^a^	129.7±51.1^c^
A1C (%)	5.9±.5^a^	7.9±2.4^b^	5.8±.4 ^a^	7.0±1.6^c^
Insulin (μIU/mL)	10.2±6.1	10.0±7.9	11.9±9.8	12.8±9.8
HOMA2-IR	1.3±.8	1.5±1.1	1.5±1.2	1.8±1.2
Log-HOMA2-IR	0.15±.5	0.17±.6	0.22±.6	0.38±.6

Note: data were expressed as mean ± SD for continuous variables or N (%) for categorical variables. Variables in groups with different superscripts are significantly different using Holm’s sequential Bonferroni post hoc test, P < .05. Abbreviations: BMI= body mass index; WC= waist circumference; HOMA2-IR=Homeostasis Model Assessment for Insulin Resistance; Hs-CRP= high sensitivity C- reactive protein; FPG= fasting plasma glucose; A1C= hemoglobin A1C; abdominal obesity male = 102 cm/ female = 88 cm); adiponectin (median- < 14.75low/ ≥ 14.75high)

**Table 4.** Association of insulin resistance with diabetes status, abdominal obesity and adiponectin by ethnicity

Ethnicity	Diabetes status	Waist circumference	Adiponectin (ng/mL)	Mean difference	SE	*P*-value	Interval difference	

							Lower bound	Upper bound

Haitian	without T2DM	normal	<14.75	−0.590	0.199	.003	−0.981	−0.198
American vs.			≥14.75	−0.093	0.152	.542	−0.391	0.206
		
African		high	<14.75	−0.023	0.171	.894	−0.359	0.314
American			≥14.75	0.061	0.154	.691	−0.242	0.364

Haitian	with T2DM	normal	<14.75	0.115	0.316	.717	−0.507	0.737
American vs.			≥14.75	−0.211	0.234	.368	−0.673	0.250
		
African		high	<14.75	−0.610	0.227	.008	−1.056	−0.163
American			≥14.75	−0.083	0.148	.577	−0.374	0.208

Abbreviations: SE= Standard error. Abdominal obesity (Waist circumference, male = 102 cm/ female = 88 cm), adiponectin (median- < 14.75 low/≥14.75 high)

**Table 5.** Association of insulin resistance with ethnicity, diabetes status, and adiponectin by abdominal obesity

Waist circumference	Ethnicity	Diabetes status	Adiponectin (ng/mL)	Mean difference	SE	*P*-value	Interval difference	

							Lower bound	Upper bound

Normal vs. high	Haitian	without T2DM	<14.75	−0.577	0.154	< .001	−0.880	−0.273
	American		≥14.75	−0.276	0.154	.074	−0.578	0.027
		
		with T2DM	<14.75	−0.283	0.194	.145	−0.664	0.098
			≥14.75	−0.409	0.193	.035	−0.788	−0.029

Normal vs. high	African	without T2DM	<14.75	−0.010	0.216	.964	−0.434	0.414
	American		≥14.75	−0.122	0.152	.424	−0.421	0.178
		
		with T2DM	<14.75	−1.007	0.334	.003	−1.664	−0.351
			≥14.75	−0.280	0.203	.170	−0.680	0.120

Abbreviations: SE= Standard error. Abdominal obesity (Waist circumference, male = 102 cm/ female = 88 cm), adiponectin (median- < 14.75 low/≥14.75 high)
